# Trauma and Coping Strategies in Police Officers: A Quantitative-Qualitative Pilot Study

**DOI:** 10.3390/ijerph18030982

**Published:** 2021-01-22

**Authors:** Cristina Civilotti, Giulia Di Fini, Daniela Acquadro Maran

**Affiliations:** Department of Psychology, Università di TorinoVia Verdi, 1010124 Torino, Italy; cristina.civilotti@unito.it (C.C.); giulia.difini@unito.it (G.D.F.)

**Keywords:** police officers, trauma, stress reactions, coping strategies

## Abstract

Background. Because of their work, emergency workers, such as police officers (POs), are exposed to traumatic events on a daily basis. These experiences can have consequences in terms of physical and emotional stress. Primary attachment relationships affect the development of coping strategies for dealing with stressful events (primarily hyperactivating strategies in entangled adults and hypo-activating strategies in dismissing adults). In this study, we explored how POs describe the experience of traumatic accidents, the effects they reported and their coping strategies related to their attachment style. Methods. We used a quantitative-qualitative method. Thirty-nine POs were administered the Beck Depression Inventory, the Maslach Burnout Inventory and a semi-structured interview about traumatic events and reactions. Interviews were analyzed using Interpretative Phenomenological Analysis. Results. Traumatic events at work predominantly concerned aggressions, witnessing deaths, forced hospitalizations, and domestic violence involving children. POs with a responsible role were more likely than POs to use security-based strategies. Most POs narrated overactivation and deactivation strategies, which were associated with depressive symptoms, emotional exhaustion, and depersonalization. Conclusions. These results can be useful to improve trauma-informed interventions for POs based on their different attachment styles and coping strategies.

## 1. Introduction

Emergency workers are exposed to traumatic events that can have consequences in terms of physical and emotional stress. Although these workers may have developed a certain degree of tolerance, due, for example, to experience, this does not mean that they do not experience the consequences of trauma [1,2,3]. Police officers (POs) also work in a context characterized by high organizational demand (e.g., responding to many tasks, from patrolling the streets to involuntary commitments, in [4]) and lack of resources (e.g., lack of social support, in [5,6,7]). In this environment they can be subject to violence as a direct result of their employment [8,9,10]. In accordance with the most recent literature [11,12,13,14,15], there is a clear need to explore and understand the nature of traumatic incidents and their impact on POs to assess the possible psychological and physiological consequences.

This does not mean that all POs perceive these events as traumatic in the same way: life experiences, especially in early childhood, the presence or absence of support mechanisms, and environmental and genetic aspects are factors that determine the response to events [16,17]. For example, although there is a high level of exposure to traumatic accidents, only a minority of these accidents are identified by POs as stressful or traumatic [18,19]. To better describe the traumatic experiences in this particular population, Mitchell [20] conceived the term “critical incident”, defined as “any situation faced by emergency service personnel that causes them to experience unusually strong emotional reactions that have the potential to interfere with their ability to function, either at the scene or later” (p. 36). Examples of accidents that fall into this category are death or serious injury of a colleague while on duty, death or serious injury of a child, threat of violence or personal injury, serious injury and death, extreme accidents and multiple deaths, feeling of helplessness in an accident, and excessive media interest in a particular case [1,8,16,18]. A qualitative study by Ricciardelli and colleagues [21] that involved 284 subjects showed that the most traumatic events were violent death, accidental death and serious road accidents. Previously, Bracken-Scally and colleagues [22], in a critical accident stress management study, found that the most common incidents for which subjects required support were witnessing someone being badly injured or killed while on duty and acts of violence or threat of violence while responding to an incident.

### Coping with Critical Incidents: The Role of Adult Attachment

Another aspect that is strongly linked to an individual′s vulnerability to traumatic events is the coping strategy adopted. Coping is defined as “cognitive and behavioral efforts to manage specific external and internal demands that are appraised as taxing or exceeding the resources of the person” [23] (p. 141). Coping strategies influence psychological well-being and are important in determining the consequences after exposure to a critical incident [24]. The literature indicates that the use of adaptive strategies is more effective in mitigating the negative consequences of trauma, while maladaptive strategies negatively affect the resilience capacity of POs [25,26].

Several studies have focused on the link between attachment and emotional well-being in facing critical accidents on military corps and public officers such as soldiers in the Gulf War [27], ambulance workers [28,29], police [30,31], firefighters [32] and nursing staff [33]. They all highlight how insecure attachment styles are associated with more acute distress after critical incidents, current emotional distress and, in the long run, more severe mental health problems, including depressive symptoms and burnout [33,34].

More specifically, Shaver and Mikulincer [35] argue that hyperactivating strategies contribute to increasing the monitoring of external threats and the feeling of unavailability of the attachment figure, aggravating the potential negative consequences of these threats and intensifying emotional responses with negative connotations. Hyperactivating strategies are associated with individuals who have an anxious attachment style, the latter being connected to an exaggerated assessment of threats, negative self-opinions and pessimistic beliefs about relationships with others [36,37,38,39,40]. Individuals with anxious attachment may react to adversities with extreme distress and may become anxious about mulling over their concern about threats [39,41]. In contrast, other individuals aim to keep the attachment system deactivated to avoid frustration and anxiety due to the perception of emotional unavailability of the attachment figure [36]. The effort to achieve autonomy and independence is therefore very high, distancing oneself physically, emotionally and cognitively from other people.

The objective of this exploratory research was to collect data on how POs describe the experience of traumatic accidents, the emotional consequences and the strategies they used to cope with these events. To better understand their strategies for dealing with the traumatic event, we used a quantitative-qualitative method. The quantitative data made it possible to collect the results of coping in terms of depressive symptoms and burn-out. For the collection of qualitative data, the chosen method was the narrative approach. In the narrative approach, attachment is considered an interpersonal motivational system that leads people to seek closeness and protection in situations of vulnerability. Through its activation the individual also develops the narrative schemes of the self and the ability to share his own stories. The concept of attachment in the strict sense was not used: to better adapt to the organizational context, the strategies for regulating emotions in relation to traumatic experiences were evaluated through interpretative phenomenological analysis.

## 2. Materials and Methods

### 2.1. Ethics and Procedure

This study was approved by the University of Torino ethics committee (protocol number: 14526). This research conforms to the provisions of the Declaration of Helsinki [42], and all ethical guidelines were followed as required for conducting human research, including adherence to the Italian legal requirements of the study and the norms of the code of ethics of the Order of Italian psychologists. Following the approval of the Ethics Committee of the University of Turin, the commander of the POs was contacted. After receiving the approval of the command, internal communication communicated participation in the study by the University of Turin. An email was sent to the institutional addresses of the POs to indicate the objectives of the research and the ethical aspects. Subsequently, arrangements were made for the administration of questionnaires and interviews. The administration period was between November 2019 and January 2020.

Participants were assured of the anonymity of their responses through fulfilment of ethical guidelines for conducting interviews. Participation in the survey was voluntary, and participants did not receive any reward for their participation.

### 2.2. Materials

The participants were given a questionnaire consisting of the following scales:

- Beck Depression Inventory (BDI II—Italian version— [43]) is a self-assessment measure of depression. The 13-item version was used. Each item is made up of 4 sentences that indicate the presence or absence of negative thoughts or feelings, and each of these sentences is assigned a value ranging from 0 to 3 [44]. For example, the first group of sentences is: (0) I do not feel sad, (1) I feel sad and melancholy, (2) I′m melancholy and sad all the time and cannot get out of it and (3) I′m so sad and unhappy that I cannot bear it. The total score can range from 0 to 39. The breakdown of scores into four different levels of presence and severity is widely recognized and used in the literature (scores of 0–9 indicate minimal depression, scores of 10–18 indicate mild depression, scores of 19–29 indicate moderate depression and scores above 30 indicate severe depression) [45]. In this study, Cronbach′s alpha was 0.68.
- Maslach Burnout Inventory (MBI—Italian version— [46]) is a scale is made up of 22 items with a Likert-type response format with the options of never (0), a few times per year or less (1), once a month or less (2), a few times per month (3), once per week (4), a few times per week (5), and every day (6). It comprises three subscales: the emotional exhaustion subscale (EE), composed of nine items and including characteristics such as fatigue and loss of energy, showing a combination of the physical and mental components of exhaustion (e.g., “I feel emotionally drained from my work”). The second, composed of five items, is labelled depersonalization (DP), manifesting negative aspects of the responses and attitudes involving other individuals and constructs such as irritability and loss of motivation (e.g., “I feel I treat some recipients as if they were impersonal objects”). The third subscale corresponds to personal accomplishment, which contains eight items (e.g., “I can easily understand how my recipients feel about things”) and measures feelings of competence and successful achievement in one′s work. In this study, Cronbach′s alpha was 0.88.

Following the questionnaire administration, in the same day, we proceeded with the administration of the in-depth interview. No participant dropped out after the questionnaire administration. The interviews were conducted by the first author, CC. The semi-structured interviews, lasting approximately 30 min, investigated the traumatic events connected to the working context with a trace that included both the analysis of potentially traumatic events attributable to routine duties (example of question and sub-question: “What events do you consider traumatic in your daily work? How do you usually react to them?”), and the events with a high traumatic impact (example of question: “Do you think about the most traumatic event that has happened to you in the workplace. Do you remember how you reacted at that moment?”). For recording the interviews, consent was obtained from all individual participants, and in case of dissent to the recording, the narrative was collected through annotations faithful to what the participant told the interviewer.

### 2.3. Textual Analysis

All recordings were transcribed verbatim and related transcripts were de-identified before analysis. All the portions of the interviews referred to the incidents described above were analyzed. To code the narrative interview, we used the Interpretative Phenomenological Analysis (IPA) method. IPA is a qualitative analysis method developed by Smith in 1990 [47], with a view to experiential psychology in clinical practice, with the aim of examining the way in which individuals attribute meaning to their experiences [48]. Using this methodology, the researcher takes an active role in the process, trying to “enter” as much as possible into the internal world of the participant. On the other hand, it is important to consider that it is not completely possible due to the researcher′s own conceptions, which invalidate the participant′s access to the world. However, the researcher′s conceptions are necessary to activate an interpretative process; at this point, it can be said that the process is based on a double hermeneutic: while the participants are trying to make sense of their world, the researcher is trying to make sense of the participants who are trying to make sense of their world [49]. The interviews were analyzed using a three-step approach [48]:

- Superficial reading of the transcripts was performed. It is advisable to read the interview several times and possibly, if available, to listen to the recording again. In this phase, the researcher can take notes and note their reflections, bearing in mind that each rereading could reveal new interpretative ideas. The researcher can focus on the content as well as on the use of language, trying to highlight the participant′s distinctive phrases or emotional responses.
- Emerging themes were identified starting from the notes taken during the interview phase. Starting from the parts of the interview that have been highlighted, we tried to formulate a sentence (subtheme) that contains the psychological meaning of that particular salient segment of the interview.
- Clustering of sub-themes was then performed. Links are sought between subthemes that are grouped under a label (theme) that provides a description of the given grouping. At this stage, all subthemes that do not fit into any of the clusters can be discarded [49].

The analytical process was independently conducted by two members of the research team who are experts in psychopathology (GDF) and in organizational psychology (DAM) (Cohen’s k before reaching an agreement = 0.85 [50]). Possible mismatches were discussed between the coders to clarify themes and to achieve a sufficient level of agreement.

### 2.4. Statistical Analysis

Statistical analyses were performed by the first author, CC, using the statistical software SPSS, version 26 9. (IBM, Armonk, NY, USA).Firstly, because of the small sample, we checked all the assumption needed to run the statistical analysis. We verified the assumption of normality through the analysis of the skewness and the kurtosis and through the Shapiro-Wilk test, explicitly recommended with small sample sizes [51].

Descriptive measures (means ± SD) were calculated for all test variables for all groups of participants. The t-test was performed to evaluate the statistical significance of EE, DP and PA subscales respect to the tool norms (*p* < 0.05 was considered to be significantly different). Because of the categorical nature of the data, χ^2^ tests were used to evaluate the cooccurrence of gender and role with regards to the coping strategies. The effect size (Cramer’s V) was calculated to estimate the practical significance of the differences. As a post hoc test, standardized Pearson residuals (SPR) were determined for each cell to determine which cell differences contributed to the χ^2^ test results. SPRs with absolute values greater than 1.96 implied that the number of cases in that cell was significantly greater than would be supposed if the null hypotheses were true (in terms of both overrepresentation or underrepresentation), with a significance level of 0.05.

Next, quantitative and qualitative data were combined, as the data derived from the textual analyses were used to create homogeneous clusters of meaning. These clusters of meaning were used as descriptors of three groups, and these groups were compared using ANOVA in relation to the parameters that emerged throughout the questionnaires (depression and burnout scores). A Tukey post hoc test was used. Estimates of effect size (Eta squared) were calculated to describe the proportion of total variability.

### 2.5. Participants

The sample was composed of 39 out of 42 municipal police POs of the Municipality of Collegno. One subject was away from work due to illness at the time of administration and two POs refused to participate. The participation was voluntary and participants did not receive any compensation. Collegno has less than 50,000 inhabitants and is located in the metropolitan area of Turin, a city in the north of the country. Socio-demographic characteristics of the sample are shown in Table 1. The weekly working hours are on average 35.42 (SD = 5.08). On average, the working age was 18.05 years (range = 1–38; SD = 11.25). The average age was 47.18 years (range = 28–62; SD = 9.71). Twenty-nine (74.4%) participants declared that they had a stable relationship, 9 (23.1%) declared that they did not have a stable relationship, and one person did not answer the question. Regarding the number of children, 15 (64.1%) indicated having children (range = 1–3); moreover, one participant out of three took care of someone permanently (e.g., an elderly parent).

## 3. Results

### 3.1. Quantitative Exploratory Analysis

The assumption of normality for BDI, EE, DP and PA scores were satisfied for all the coping styles groups, as assessed by Shapiro-Wilk′s test (*p* > 0.05). Because there was a small-medium effect size (the η^2^ range was comprised between 0.08 and 0.37) the required sample size would be *n* = 159. As such, the outcome of the ANOVA should be interpreted with caution, as the results might be different with a larger sample size. Average scores for depressive symptoms and burnout are shown in Table 2. From the analysis of the questionnaire data, it emerged that 31 POs (79.5%) reported a minimal level of depression, 7 (17.9%) reported mild depression, and only one (2.6%) reported a moderate level of depression, while no participants exhibited severe depression. Comparing the POs of this sample with the normative scores in the Italian context [52], they have a comparable level of emotional exhaustion (EE), while they have a higher level of depersonalization (M = 10.23 SD = 6.56 vs. M = 5.34 SD = 5.44, *t* = 5.53, *p* < 0.01) and lower level of personal accomplishment (M = 30.67 SD = 6.46 vs. M = 33.99 SD = 8.38, *t* = 2.49, *p* = 0.01).

### 3.2. Qualitative Analyses: Causes of Police Trauma and Prevalence of the Use of Safe, Hyperactivating and Deactivating Strategies in POs

From the analysis of the transcripts, it appears that the traumas indicated by the POs participating in the research refer to the aggression suffered (*n* = 15), to intervention in the event of death on the street or home (*n* = 12), to the forced hospitalization of subjects with psychopathologies (*n* = 5), and to interventions for domestic violence involving children (*n* = 3). Two subjects indicated excessive exposure to the media as a traumatic event, while one subject described the murder/suicide of a colleague, which occurred in another command, as a traumatic event. Six subjects identified more than one traumatic event.

Using the IPA methodology, the transcripts were interpreted with the aim of investigating the various trauma processing management strategies of the interviewees. The parts of the transcripts that involved critical incidents were highlighted and analyzed by formulating subthemes; subsequently, the subthemes were clustered within themes. Table 3 shows the results of the application of the IPA methodology to the transcripts of the interviews, where the column “textual units” shows some parts of the interview (examples) that have been highlighted by the analysis, and in the other columns the sub-themes and themes corresponding to the parts of the transcript were considered, respectively. In 17 of the 39 transcripts, the following subthemes emerged: overwhelmed, rumination, fragile and negative self-opinion, anger (high reactions to distress), and helplessness. The subjects who predominantly used these strategies were grouped into those who used hyperactivating strategies.

In 10 transcripts, the subthemes that emerged were research/need for autonomy, repression/denial of negative emotions, lack of memory/lack of access to traumatic events, and distancing/disengagement. Subjects who primarily used these subthemes were included in the group of those who mainly used deactivating strategies. The remaining 12 transcripts generally presented an optimistic view of conflict management, a positive view of oneself and of the other, a higher sense of autonomy and self-efficacy and the absence of defense mechanisms. Security-based strategies have been attributed to these subjects [40].

### 3.3. Convergence of Quantitative and Qualitative Analyses

Trauma processing strategies were the subject of a subsequent analysis that aimed to highlight any statistically significant differences between the use of the three strategies that emerged from the textual analysis (free, hypo-activating or hyperactivating) and the socio-demographic variables gender, age, and role (Table 4). Additionally in this case, due to the small number of subjects, results should be interpreted prudently because there is a violation of the assumptions for a Chi square test and not all the cells are larger than *n* = 5. It should be noted in particular that those who have a role of responsibility appear to adopt a defensive strategy against trauma of a safe type more than POs (|SPR| = 1.98, Cramer′s V = 0.15). Regarding age, the t-test did not show statistically significant differences between the average age and the use of trauma coping strategies (*t* = −0.51; *p* = 0.359).

The ANOVA test highlights differences between groups in experiencing depressive symptoms and burnout (Table 5). Significantly different means are indicated with different letters at the apex. There was a statistically significant difference between groups as determined by one-way ANOVA on the EE BMI subscale (F (2,38) = 10.53, *p* < 0.001) and the DP subscale (F (2,38) = 7.05, *p* = 0.003). The Tukey post hoc test revealed that both hypo- and hyperactivating subjects exhibited greater scores on the EE and DP subscales compared to subjects who primarily used secure strategies. There was no significant difference between groups on the PA subscale. Regarding BDI, a statistically significant difference was found between groups (F (2,38) = 7.80, *p* = 0.002). The Tukey post hoc test revealed that both hypo- and hyperactivating subjects presented higher levels of depression than subjects who primarily used secure strategies.

## 4. Discussion

The goal of this pilot research was to examine trauma management strategies based on safety and insecurity in POs. A generalization of the results is not warranted, due to the small sample involved. The first interesting result was related to the type of trauma indicated by the participants. In line with what emerged from the research of the authors [53], in this case, the data indicate that trauma in this population was primary linked to aggressions, intervention in case of death, and to interventions for domestic violence involving children. The intervention for forced hospitalization of subjects with psychopathologies was interesting. As indicated by Wood and colleagues [54], POs are often not trained for interventions on subjects with psychopathologies. Consequently, the intervention risks are more difficult for both OPs and subjects who need to be hospitalized. As suggested by Puntis and colleagues [55], a functional intervention model can be one in which the intervention unit is made up of a PO and a clinician. This collaboration can mitigate the response of the POs, for example, when faced with a behavior whose motivations they do not understand, they may react in an inappropriate way (for example, by exacerbating the subject′s fear and in fact triggering an escalation of violent behavior). Compared to narrative information, these findings made it possible to obtain information on how the subject manages his own and others′ vulnerability and regulates himself in response to a trauma [35]. Moreover, these findings could be useful to better understand problems that are typically related to POs’ work [56]. Results relating to the strategies implemented in the face of a traumatic event indicate that managers of the PO tend to use strategies based on safety more than the POs. These subjects are able to maintain close ties with others, relieving discomfort and enhancing personal adaptation through constructive, flexible and congruent mechanisms with reality. The positive experience also has the effect of expanding the resources to face challenging emotional situations not only related to the trauma but also to the stress that the nature of this work inevitably brings with it [31]. On the interpersonal level, positive experiences based on safety promote constructive relationship styles in social situations and the ability to develop resilience. Most POs, particularly agents, use overactivation and deactivation strategies. Such strategies may result in depressive symptoms, emotional exhaustion, and depersonalization. As described by Basinska, Wiciak and Daderman [57], negative emotions in POs increase the feeling of fatigue and disengagement, resulting in low motivation to work and reducing the ability to work. For this, it is important that training programs for POs are also focused on emotion-regulation skills to prevent mental health problems in this population [58].

The hyperactivation strategy adopted by the POs involves exaggerating the perception of the severity of the danger and the implementation of a state of perennial vigilance. This strategy may lead the person to perceive minimal signs of disapproval, decreased interest or imminent abandonment. POs who adopt these strategies can have easy access to thoughts and emotions related to threatening situations and have difficulty keeping them under control. Furthermore, it can be easy for them to maintain contradictory cognitions of the past and present negative experiences in their working memory by mixing them together, creating a chaotic mental situation dominated by negative emotions (lack of trust in others, anger, use of passivity). POs who use deactivation strategies, on an interpersonal level, strategically try to live with detachment to exercise control and leverage themselves [30]. These subjects may avoid directly and symbolically facing tensions and relational conflicts; they can have little willingness to face discomfort and a lack of need for closeness and safety on the part of others. They may also tend to experience detachment, cold and superficial interpersonal relationships, not to face relationship problems and to leave conflicts unresolved.

In this work, our ultimate clinical aim was to give a contribution in tailoring efficient interventions for deal with trauma in POs. Before discussing which strategies and intervention plans can be used with this population, it is useful to note that no single strategy has proven most suitable for addressing this complex phenomenon. However, each context must be thoroughly examined to outline a possible intervention that can be conducted effectively. Therefore, each situation requires ad hoc treatment that accounts for all variables involved in the phenomenon.

Although this project has an exploratory perspective, our indication is to provide, when possible, a context analysis to understand the emotional climate and the coping strategies of the POs involved in a critical incident. In fact, different stress management strategies emerge from the research and the same intervention can be useful for some subjects and detrimental for others.

Scientific and clinical projects are going in that direction. For example, several study show how a debriefing intervention, after each traumatic event, can facilitate the processing of trauma [59,60], just as a training intervention and taking charge of possible trauma outcomes could help mitigate its effects. On an individual level, for example, Arnetz and colleagues [61] proposed improving the feeling of control of POs in dealing with traumatic events by providing a list of useful strategies based on the type of event experienced. Similarly, Papazoglou and Andersen [62] devised a program that improves resilience in POs by learning the use of adaptive strategies. Svetlitzky and colleagues [63] also proposed teaching POs techniques of concentration on action to increase the control of emotions in the face of a traumatic event. Wilson and colleagues [64] used Eyes Movement Desensitization and Reprocessing and found that POs have lower ratings on measures of PTSD symptoms, subjective distress, job stress, and anger, even after 6 months of follow-up. Anshel and colleagues [65], alongside the teaching of adaptive strategies, proposed that POs include physical activity programs. In the meta-analysis conducted by Purba and Demou [66], the importance of support for POs by the organization emerged, in particular from superiors and colleagues, as positive feedback is linked to improved mental health outcomes. Furthermore, from an organizational point of view, training courses on the consequences of the use of strategies in dealing with a traumatic event, the construction of an organizational climate capable of supporting the POs and allowing the expression of negative emotions can be useful [67]. Above all, as suggested by Salston and Figley [68], the possibility of having the supervision of a clinician who activates debriefing techniques following the traumatic event is of fundamental importance. These techniques allow one to deal with both the traumatic event and the emotions connected to it, helping to manage stress within and outside the professional context.

### Limitations

This study opens up many questions on the prevention and management policies of work-related stress in POs, but it obviously has limitations. First, the sample number was small and the study was underpowered: for this reason, results must be interpreted with caution, as the outcomes might be different with an adequate sample size. Subjects who participated in this study worked in the same location, sharing traumatic events. This sharing can lead to a mechanism that allows you not to let go of the memory because the effects of the trauma are visible and occur in every day [69,70]. Another limitation is related to the use of the qualitative methodology. As indicated by several authors (e.g., [71,72]), the limit of quantitative-qualitative research lies in the impossibility of generalizing the data, describing the results within that specific group of subjects. It is also true that, as indicated by Ochieng [73] and de Souza Minayo [74], the wealth of quantitative and qualitative data allows for reflections and ideas for further analyses that can be conducted with different methods. Future research may be conducted based on the data obtained here to better understand the link between coping strategies adopted in the case of traumatic events and attachment styles in POs.

## 5. Conclusions

The data obtained herein can be used to better implement the method of selection of POs, the training dedicated to them at the moment of entry and supervision during the course of their work. In light of what has emerged, the hope is that intervention in cases of traumatic events can be done on the basis of the different attachment styles that characterize POs. This will improve the quality of life of the POs and allow adoption of adaptive strategies with positive repercussions in the work environment as well as the home environment.

## Figures and Tables

**Table 1:** Socio-demographic characteristics of the sample (*n* = 39).

	*n*	%
Sex:		
- Male	27	69.2
- Female	12	30.8
Sector:		
- Operative	31	79.5
- Administrative	7	17.9
Shift work:		
- Day shift	27	69.2
- 24-h shift	9	23.1
- None	1	2.6
Role:		
- Patrol Police Officer	29	74.4
- Team Manager	7	17.9
- Organizational Position	1	2.6

**Table 2:** Average scores for depressive symptoms and burnout (*n* = 39).

	M	SD	Min.-Max.
BDI	6.25	5.04	0–19
MBI (EE)	22.45	10.31	6–41
MBI (DP)	10.23	6.55	0–27
MBI (PA)	30.66	6.46	1–42

Note. M = Mean; S.D. = standard deviation; Min.-Max = range of scores.

**Table 3:** Textual units, subthemes and themes. Examples from interviews (*n* = 39).

Textual Units	Sub-Themes	Themes
“Well, sometimes it is not really possible, because the amount of data is such, it is a lot… sometimes we have the radio calling, the telephone ringing, Carabinieri who come to ask you how the images are now, that is, you really can′t to cope with all this input that comes at the same time. So, the only thing to do is try to disconnect for a moment and try to pass, perhaps, one input at a time if possible, if they are not really closely connected, of course.” “It′s all too much. At some point we should start filtering upstream, so that all of us who are in front live are not compressed and crushed by a flood of data”.	Feeling of being overwhelmed/overwhelmed (overwhelming)	Hyperactivating strategies
“When you try to be proactive, but there are people who block everything, I don′t care about order and degree, in fact nothing is done. How you do it, you get it wrong. So, you don′t know what to do “. “There was my commissioner who gave the cardiac message and here I felt helpless and said ′but what are you doing to him, if he′s lost fluids he′s dead, he′s gone′…”	Impotence	
“They use any kind of detail (error, procedure, anything, barely interpretable rules), they use this as a lever, they disintegrate you and when you are pulverized, they pass over you. I′m referring to internal people, officers”	Anger	
“Also, because when the mess happens then you are responsible, and here comes the speech of the top management. … I can′t take it anymore...we are not recognized. If you can′t escape, I adapt, but the future isn′t too bright” “When you see that it is being belittled... I am unable to withstand the confrontation with the people I know, who... I have consideration, I am unable to withstand the trial…”	Fragile/negative opinion of oneself	
“The most traumatic experience, because it was the first left... I don′t know if because visually, because of the smell or what, but I still remember this... in talking about it I am living it, I really live it, I am anxiety”	Rumination	
“… Be colder with people and… and try to detach yourself from problems, because otherwise you take them home and then you don′t even live anymore. And so, the people next to you are also not well”.	Disengagement	Deactivating strategies
“But like a bit of all things, when you do something it bothers me to make a fool of myself, and-well, one thing that could create stress for me is that I have to make a fool of myself, then I want to avoid this stress and before make a fool, I prepare myself so as not to make a fool”	Research/need for autonomy	
“Eh…nothing happened…” “It didn′t happen… I don′t remember…”	Lack of memory	
“I don′t have great sources of stress, in the sense not if we mean stress what creates discomfort. If-if we mean stress in the sense…that…you realize that you have to commit yourself, that yes”	Suppression of negative emotions	

**Table 4:** Sociodemographic characteristics of the participants who used different strategies to cope with the trauma. Values are expressed as *n* (percentage) (*n* = 39).

	Free	Hyperactivating	Hypo-Activating	χ^2^	*p*
Gender:					
- Male	10 (25.6)	7 (17.9)	10 (25.6)	1.99	n.s.
- Female	2 (5.1)	3 (7.7)	7 (17.9)		
Role:				15.81	0.015
- Agent	5 (12.8)	10 (23.1)	15 (38.5)		
- Person in Charge	5 (12.8)	-	2 (5.1)		
- Other	2 (5.1)	-	-		

Note: χ^2^ = chi square value; *p* = *p*-value; n.s. = not statistically significant.

**Table 5:** Depression and burnout symptoms: comparison among free, hypo-activating and hyperactivating methods (one-way ANOVA) POs (*n* = 39).

	Free (*n* = 12) M(SD)	Hyperactivating (*n* = 17) M(SD)	Hypo-Activating (*n* = 10) M(SD)	*F*	*p*	*η^2^*
BDI	3.44 (2.74) ^a^	9.33 (5.26) ^ab^	4.38 (4.05) ^b^	7.80	0.002	0.32
MBI (EE)	16.56 (8.54) ^a^	29.47 (8.09) ^ab^	17.60 (8.80) ^b^	10.53	0.000	0.37
MBI (DP)	6.75 (5.51) ^a^	14.11 (6.53) ^ab^	7.82 (4.14) ^b^	7.05	0.003	0.28
MBI (PA)	32.50 (5.13)	28.67 (7.83)	31.85 (4.66)	1.50	n.s.	0.08

Notes: M, mean; SD, standard deviation; F, Fisher’s ratio; *p* = *p*-value; n.s., not statistically significant¸ *η^2^* = effect size. Significantly different means are indicated with different superscript letters ^a, b, ab^.
